# Measuring skin necrosis in a randomised controlled feasibility trial of heat preconditioning on wound healing after reconstructive breast surgery: study protocol and statistical analysis plan for the PREHEAT trial

**DOI:** 10.1186/s40814-017-0223-y

**Published:** 2018-01-17

**Authors:** Suzie Cro, Saahil Mehta, Jian Farhadi, Billie Coomber, Victoria Cornelius

**Affiliations:** 1 Imperial Clinical Trials Unit, School of Public Health, Imperial College London, London, UK; 2 Guy’s and St. Thomas’ NHS Foundation Trust, London, UK

**Keywords:** Feasibility study, Breast cancer, Mastectomy, Breast reconstruction, Heat preconditioning, Necrosis, Statistical analysis plan, Outcome selection

## Abstract

**Background:**

Essential strategies are needed to help reduce the number of post-operative complications and associated costs for breast cancer patients undergoing reconstructive breast surgery. Evidence suggests that local heat preconditioning could help improve the provision of this procedure by reducing skin necrosis. Before testing the effectiveness of heat preconditioning in a definitive randomised controlled trial (RCT), we must first establish the best way to measure skin necrosis and estimate the event rate using this definition.

**Methods:**

PREHEAT is a single-blind randomised controlled feasibility trial comparing local heat preconditioning, using a hot water bottle, against standard care on skin necrosis among breast cancer patients undergoing reconstructive breast surgery. The primary objective of this study is to determine the best way to measure skin necrosis and to estimate the event rate using this definition in each trial arm. Secondary feasibility objectives include estimating recruitment and 30 day follow-up retention rates, levels of compliance with the heating protocol, length of stay in hospital and the rates of surgical versus conservative management of skin necrosis. The information from these objectives will inform the design of a larger definitive effectiveness and cost-effectiveness RCT.

**Discussion:**

This article describes the PREHEAT trial protocol and detailed statistical analysis plan, which includes the pre-specified criteria and process for establishing the best way to measure necrosis. This study will provide the evidence needed to establish the best way to measure skin necrosis, to use as the primary outcome in a future RCT to definitively test the effectiveness of local heat preconditioning. The pre-specified statistical analysis plan, developed prior to unblinded data extraction, sets out the analysis strategy and a comparative framework to support a committee evaluation of skin necrosis measurements. It will increase the transparency of the data analysis for the PREHEAT trial.

**Trial registration:**

ISRCTN ISRCTN15744669. Registered 25 February 2015

**Electronic supplementary material:**

The online version of this article (10.1186/s40814-017-0223-y) contains supplementary material, which is available to authorized users.

## Background

Although the majority of women with breast cancer are treated with breast conserving surgery, approximately 30% will still need a mastectomy. The 2002 National Institute for Clinical Excellence (NICE) guidelines recommend immediate breast reconstruction whenever it is safe [1]. Revised NICE guidance in 2009 re-emphasises the importance of immediate reconstruction after mastectomy [2].

There are essentially two types of reconstruction that a patient can have after mastectomy: implant-based or autologous flaps, where the patient’s own tissue is used to create a new breast. Skin-sparing mastectomy (SSM) followed by autologous microsurgical breast reconstruction is an increasingly popular procedure within the UK [3]. It results in highly satisfactory results both oncologically and cosmetically from a surgical and patient’s perspective [4]. In 2011, 16,485 women underwent mastectomy in the UK and approximately a third of these underwent autologous reconstruction [5].

These procedures, particularly autologous reconstruction, are highly labour intensive requiring significant expertise and involvement of several healthcare professionals. Implant-based reconstruction is less expensive initially than autologous reconstruction but more patients are opting for the latter due to more satisfactory results [6]. A bilateral mastectomy and reconstruction can take an entire day of operating theatre time. The average cost of a free flap breast reconstruction is estimated at £10,910 compared to an implant only reconstruction, which costs £8034 [6]. However, it is important to remember that autologous reconstruction has a lower long-term cost to the NHS as it requires fewer revisions and has higher patient satisfaction compared to implant reconstruction [7].

According to the NHS’ Institute for Innovation and Improvement, reducing the length of hospital stay is ranked as a level 1 priority [8]. In autologous breast surgery, the average length of stay (LOS) following a “deep inferior epigastric perforator” (DIEP) flap is 8 days and our own LOS concurs. In the USA, the LOS is much shorter however this is mainly due to the reluctant nature of private funding from insurance companies for the same procedure [9]. LOS is the most influential factor in reducing the cost of autologous breast reconstruction [9]. Local heat preconditioning could help improve the provision of this procedure by helping to reduce the complications and overall costs associated with its service delivery.

### Evidence for local heat precondition

A non-randomised phase 1 study conducted by three of the authors (JF, SM and VC) in 50 participants looked at the use of heat preconditioning to reduce skin necrosis in SSM. The study showed a reduction in necrosis from 36 to 12% (controls vs tests) and an average length of stay from 8 to 4 days [10]. There is little data on the cost implications of specifically skin necrosis, however, the incidence rate is between 20 and 40% and the cost of surgical treatment (debridement and skin graft) is approximately £1782 plus extra theatre time (£3840 for half a day in theatre [11]). Extra hospital stay means the costs can spiral into the thousands.

The simple heat preconditioning intervention we are exploring has the potential to improve wound healing preoperatively and to prevent complications in a safe way reducing the burden to patients at minimal cost. This is important in a modern NHS with increasing financial constraints and, if found to be efficacious, heat preconditioning could allow us to offer autologous reconstruction to more patients. Aside from the financial benefits, benefits to patients would be improved wound healing resulting in quicker recovery and reduced LOS with faster progression to adjuvant therapy. This means a quicker return to normal life but more importantly, earlier discharge improves recovery, reduces the risks of hospital-acquired infections and venous thromboembolism [8].

### Study aim

PREHEAT is a feasibility study for a trial that will evaluate local heat preconditioning with respect to its effects on wound healing after reconstructive breast surgery in patients with breast cancer. The aim of this study is to inform the design of a larger trial among breast cancer patients undergoing SSM and nipple-sparing mastectomy (NSM) focusing on effectiveness. This article describes the PREHEAT trial protocol (version 1.1, 26/02/2016) and the detailed statistical analysis plan, which has been developed prior to unblinded data extraction to meet the trials objectives. The protocol was prepared in accordance with the Standard Protocol Items: Recommendations for Interventional Trials (SPIRIT) guidance [12]. The trial SPIRIT checklist can be viewed in Additional file 1.

### Objectives

#### Primary objective

The primary objective of PREHEAT is to identify the best way to measure a skin necrosis and to estimate the necrosis event rate in the treatment and control arm using this definition.

#### Secondary objectives

Secondary objectives are the following:

i. To estimate the recruitment rate
ii. To estimate 30-day follow-up retention
iii. To assess the level of adherence with the heating protocol
iv. To estimate the effect of heat preconditioning on length of hospital stay
v. To estimate the rates of surgical versus conservative management of skin necrosis

The information gathered from this study will be used to inform the design of a large multicenter effectiveness and cost effectiveness trial.

## Methods/design

PREHEAT is a randomised two-arm single-blind parallel group controlled trial of local heat preconditioning in breast cancer patients undergoing SSM and NSM. The comparator intervention is mastectomy only without any heat preconditioning. Surgeons and assessors evaluating necrosis outcome will be blind to treatment allocation. The aim is to recruit approximately 180 patients over a fixed 2-year recruitment period.

### Study population

The study population will be women over the age of 18 (no maximum age limit) undergoing SSM or NSM mastectomy and immediate breast reconstruction (autologous and implant). This will include diabetics, smokers and BRCA carrier prophylactic mastectomies with immediate breast reconstruction. Exclusion criteria include delayed (2-stage) reconstruction patients, patients with a latex allergy and patients with inflammatory cancer.

### Intervention

The intervention is heat preconditioning of the breast (to be operated on) the night prior to the surgery by means of a hot water bottle. Participants in the intervention arm will be given a hot water bottle and an underwater thermometer for use at home. The preconditioning heating protocol is as follows: the participants will need to heat water in a saucepan at home to 43 °C and pour it into the bottle. This is then placed on the naked study breast (the breast to be operated on) over the nipple-areola complex for 30 min. The breast is then allowed to cool for 30 min to body temperature. The participants will then need to do another 30-min application of heat at the same temperature for 30 min with fresh heated water. The bottle must be placed in the same area. After another 30-min break, the participants will need to do a final, third heat application. The preconditioning intervention will be undertaken once, 12 h before surgery, i.e. the evening before. For the BRCA carrier status participant undergoing bilateral mastectomy and reconstruction, we will ask them to heat only the right breast.

This heating procedure is based on the protocol used in our phase 1 study and an experimental animal model investigating the effect of local heat preconditioning [10, 13]. A temperature of 43 °C provides heat application to a supraphysiological level without causing a burn. We will ask participants to record the temperature of the water and the number of heating applications they undertook to assess adherence to the heating protocol. All usual medication will be continued.

### Randomisation and blinding

Participants will be allocated to treatment arm via an online randomisation system hosted by the King’s College London Clinical Trials Unit (KCTU) to ensure concealment of treatment allocation for clinicians who are assessing participants. Randomisation is undertaken with equal allocation to each arm (1:1) using minimisation stratifying for the following:

- Type of reconstruction (Implant vs Autologous)
- Smoking status (yes/no)
- Diabetic (yes/no)
- BRCA carrier status (yes/no)

Surgeons and outcome assessors will be blind to treatment allocation.

### Outcomes

#### Primary outcome

Necrosis will be measured in three different ways as follows:

1. Necrosis (yes/no): by clinical judgement
2. Necrosis depth (assessed using the SKIN score as described below): recorded independently by two clinical outcome assessors and from photographs by two further assessors.
3. Necrosis area (mm^2^): recorded by depth using a transparent grid on day of occurrence independently by two clinical outcome assessor when necrosis is present.

Necrosis depth was initially recorded as none/superficial/partial/full/superficial + partial/full thickness during the first part of the PREHEAT study (for patients 1 to 34). Following publication of a validated SKIN score by Lemaine et al. 2015 [14], a protocol update was made in February 2016 and the SKIN score replaced the original recorded categories. The SKIN score classifies the depth of necrosis from A to D where A is none; B is colour change of flap suggesting impaired perfusion or ischaemic injury; C is partial thickness skin flap necrosis resulting in breakdown of the wound; D is full thickness skin flap necrosis [14]. All outcome assessors will be provided with training in the assessment protocol, which uses previously published and accepted methods of measuring wound area.

#### Secondary outcomes

Secondary outcomes include:

- Recruitment rate
- 30 day follow-up retention proportion
- Level of adherence with heating protocol
- Length of stay in hospital
- Rates of surgical/conservative management of skin necrosis

#### Harm outcomes

The safety of the heating procedure will be recorded first by the participant as they conduct the procedure at home and second by the surgical team on the day of surgery. Information on adverse events will continue to be collected by means of spontaneous reports from patients. Attribution of the adverse event to the intervention will be made by participant and clinical opinion.

### Frequency and duration of follow-up

The schedule of trial enrolment, interventions and assessments are presented in Table 1. Following the day of surgery monitoring for necrosis will take place on the ward as an inpatient and in outpatient clinics by a member of the clinical team blinded to intervention arm. The follow-up ends at 30–40 days post operation. To establish the reliability of the necrosis outcomes, a second individual from the clinical team will independently assess necrosis in clinic. Photographs will be taken so the necrosis depth can be assessed by two further blinded independent raters.

**Table 1:** Schedule of enrolment, interventions and assessments for PREHEAT

	Study period				
	First clinic appointment with reconstructive surgical team	Pre-assessment for surgery	Day before surgery—treatment period	Day of surgery	Follow-up
Action	Day 14	Day 7	Day 1	Day 0	Days 0–30
Eligibility screen	X				
Patient information	X				
Informed consent		X			
Randomisation		X			
Equipment given to patient		X*			
Baseline and pre-operative assessment/variables collected (see Table 2)		X			
Heat preconditioning procedure			X*		
Surgery				X	
Operative assessment/variables collected (see Table 2)				X	
Monitoring for skin necrosis and other post-operative outcomes (see Table 2)					X†
AE monitoring			X*	X	X

*For patients randomised to intervention only

†Skin necrosis measurements are taken at the first outpatient appointment (usually 7 days after discharge so approximately day 12–16) then at every outpatient visit following this until day 30–40

### Data collection

Data is collected from clinical proformas, clinical notes and study-specific forms and then entered onto an online data and management system (MACRO by InferMed (https://www.elsevier.com/solutions/macro)). The database has been programmed by KCTU and is hosted on a dedicated secure server within Kings College London. No identifiable data will be entered on the database. Participants will be identified using a unique code and initials.

### Trial Steering Committee

The Trial Steering Committee (TSC) will include an independent chair, two independent members, an independent patient representative and at least one study statistician. The committee will meet with the research team bi-annually to monitor and supervise the progress of the trial, accumulating safety data and other sources of relevant information.

### Sample size and recruitment

The main aim of the study is to identify a suitable way to measure necrosis and estimate the event rate in each arm. We also aim to estimate recruitment and retention rates and explore the potential impact on the outcome by stratification factors. In order to do this with sufficient precision to inform the design of a later definitive study, the sample size calculations have been based on estimating with precision and not hypothesis testing.

The study will take place at Guy’s and St Thomas’ NHS Foundation Trust (GSTT) in the department of plastic surgery. Within GSTT over 150 SSMs are performed per year and recruitment will occur for 2 years. Three hundred participants would allow us to estimate the 95% confidence intervals (CIs) for the recruitment rate with precision of at least ± 6 percentage points (calculation based on proportion requiring the largest sample size, e.g. 50%). Based on our previous experience, we anticipate that the actual recruitment rate will be no less than 60% so this would provide us with at least 180 participants. Assuming the proportion of necrosis events among participants in the control arm is 30%, this number will allow us to estimate ‘necrosis’ rates to within each arm to at least ± 9 percentage points. If the true difference between event rates were 15%, we will be able to estimate this within ± 12 percentage points.

We anticipate that the four consultant plastic surgeons will perform at least 30 operations each during this period (approximately 15 per arm). This sample size will also allow us to estimate means and 95% CIs for secondary outcomes and will provide an adequate number of participants in subsets (smokers, diabetics and radiotherapy patients) in order to be able to estimate the standard deviation for the continuous outcomes.

Patients will be recruited from the outpatient clinic ideally at their first appointment with the surgical team at GSTT. The second point of recruitment will be at a patient’s pre-assessment clinic if they have not been asked at their initial appointment.

### Statistical methods

#### Analysis principles

The analysis will be conducted in accordance with a pre-defined statistician analysis plan, which is described in detail below. As this is a feasibility study, the analysis for this trial will be primarily descriptive. Summary statistics will be calculated to assess feasibility aims such as the measurement of necrosis, recruitment and retention rates, surgical versus conservative management of skin necrosis and acceptability measures for patients.

Data will be analysed on an intention-to-treat (ITT) principle (i.e. all participants with a recorded outcome will be included in the analysis and will be analysed according to the treatment group to which they were randomised.) No imputation for missing data will be performed. The proportion of missing data for each outcome will be reported. Confidence intervals (CI) will be 2-sided and at the 95% level. All data will be analysed using Stata/IC (StataCorp, College Station. TX, USA).

### Analysis plan

#### Baseline comparability of randomised groups and flow of patients

A Consolidated Standards of Reporting Trials (CONSORT) Extension to Pilot and Feasibility Trials checklist flow chart will be constructed [15, 16]. This will include the number of patients screened, number of eligible patients, number of patients randomised, number of patients withdrawing and lost to follow-up, the number continuing through the trial and the number included in the analyses. Baseline characteristics listed in Table 2 will be summarised by treatment group using suitable measures of central tendencies (mean and median) and variability (standard deviation—SD and interquartile range—IQR) for continuous data, and frequencies and proportions for categorical data. No significance testing will be undertaken.

**Table 2:** Baseline/pre-operative/operative/post-operative variables

Baseline variables:
Age (years), ethnicity, height, weight, diabetic, hypertensive, smoker, smoking history, BRCA carrier, neoadjuvant therapy, previous breast surgery on study breast, type of previous breast surgery, oncological reason for mastectomy, prophylactic reason for mastectomy.
Pre-operative variables:
Pre-operative variables: measurement notch to nipple, inframammary fold to nipple, breast cup size, sensory changes, sensory changes due to, degree of breast ptosis, type of reconstruction (implant/autologous based) study breast.
Operative variables:
Surgery (yes/no), reason for no surgery if no, breast surgeon, plastic surgeon, side of study breast, type of construction, type of mastectomy, surgical technique for mastectomy, infiltration used, type of breast reconstruction, axillary clearance, core temperature pre first incision, environmental temperature pre first incision, mastectomy skin flap thickness (for upper outer, lower outer, lower inner, upper inner quadrants), mastectomy skin flap thickness measurement method, mastectomy breast weight, reconstruction flap weight, implant/expander volume, type of implant used, core temperature post reconstruction, environmental temperature post reconstruction, number of drains, duration of operation, intra-operative complications.
Additional post-operative outcomes:
Wound infection additional antibiotic course, type of antibiotic given, post-operative seroma, seroma required draining, site drainage of the seroma performed, method to detect seroma, post-operative haematoma required surgery, surgical intervention for complications of reconstruction, surgical re-intervention for reconstruction complication, repeated surgical interventions.

#### Pre-operative and operative characteristics by randomised group

Pre-operative and operative variables listed in Table 2 will be summarised by treatment group using suitable measures of central tendencies and variability for continuous data and frequencies and proportions for categorical data. No significance testing will be undertaken.

#### Analysis of primary outcome

As the anticipated number of events (necrosis yes/no) is expected to be relatively few (approximately 40 events in total: 30% control; 15% in intervention), the unadjusted proportion of participants with skin necrosis within 30 days in each trial arm with corresponding 95% CI will initially be calculated. Results will then be tabulated by treatment group for minimisation variables, smoking (yes/no), diabetic (yes/no), autologous- or implant-based reconstruction (yes/no), BRCA carrier status (yes/no).

For necrosis depth (SKIN score), we will present the unadjusted proportions of patients within each category by treatment arm, as assessed by the primary clinical assessor at time of first occurrence (or assessment if none throughout). For patients with necrosis, we will present measures of central tendencies (mean and median) and variability (standard deviation (SD) and interquartile range (IQR)) for necrosis area (3), measured in mm^2^, by treatment arm as assessed by the primary clinical assessor at time of first occurrence. Area will be reported overall (sum of areas by depth) and separately by depth grading.

Necrosis depth (SKIN score) and area measurements will be plotted over time for each individual by treatment group. We will examine the area assessments over time with a view to considering the area under the curve (AUC) as an additional summary estimate. For each patient, we will also determine the maximum depth and area as assessed by the primary assessor over the follow-up period and present the unadjusted proportions of patients within each category. For maximum necrosis area, we will present mean, median, SD and IQR by treatment arm. The proportion of patients for whom the necrosis has been resolved/fully healed within the 30 days will be presented by treatment arm with 95% CI.

The agreement between the primary and secondary assessors clinical assessment of necrosis presence (yes/no) and depth (SKIN score) will be assessed using Fleiss’ kappa statistic, κ [17]. For each patient who experiences necrosis, we will include the first occasion where necrosis has been indicated by at least one of the two assessors, i.e. at the time of first appearance. For patients who do not experience necrosis during the follow-up period, we will include the first occasion where both assessors rate the depth as none. Fleiss’ kappa statistic is appropriate since each patient is rated by the same number of assessors (*n* = 2), but the assessors are not necessarily the same over all patients. Kappa values can vary from − 1 (complete disagreement) through 0 (chance agreement) to + 1 (complete agreement). Intermediate kappa values will be interpreted using the criteria described by Landis and Koch [18] where κ < 0 = poor agreement, 0 < κ ≤ 0.20 = slight agreement, 0.20 < κ ≤ 0.40 = fair agreement, 0.40 < κ ≤ 0.60 = moderate agreement, 0.60 < κ ≤ 0.80 = substantial agreement and 0.80 < κ < 1 = almost perfect agreement.

The presence (yes/no) and depth (SKIN score) agreement for necrosis will be assessed using Fleiss’ kappa statistic between the two photographic assessors, the primary clinical assessor and photographic assessor 1 and the primary clinical assessor and photographic assessor 2.

The agreement between the first and second assessors clinical assessment of total necrosis area will be assessed using the method of Bland and Altman [19]. That is, we will visually inspect a plot of the difference between the two assessors for each assessment measuring the same quantity, i.e. for the same patient and same time, against their mean and present 95% limits of agreement calculated as d ± 1.96 s, where d denotes the mean difference and s denotes the standard deviation of the differences. The limits of agreement represent the region in which 95% of differences will lie (assuming normality of differences).

The degree of absolute agreement among area measurements will also be measured using an intraclass correlation coefficient (ICC). This will be estimated from a one-way random effects ANOVA model as different assessors are used across patients, i.e. the two assessments do not differ in a consistent way [20]. For the recorded area (in mm^2^) for patient *i* assessed by assessor *j*, denoted as *y*_*ij*_ the model to be fitted therefore will be the following:

$$ {y}_{ij}={\beta}_0+{r}_i+{w}_{ij} $$

for, *i* = 1,…,*n* patients, *j* = 1,…,2 assessors, *β*_0_: overall mean of the assessor ratings, ${r}_i\sim N\left(0,{\sigma}_r^2\right),$ and ${w}_{ij}\sim N\left(0,{\sigma}_w^2\right).$ ${\sigma}_r^2$ is the variability across patients and ${\sigma}_w^2$ is the unexplained variability across the assessments for the same patient. The ICC which represents the extent to which there is absolute agreement among the assessors is estimated as

$$ \mathrm{ICC}=\frac{\sigma_r^2}{\sigma_r^2+{\sigma}_w^2}. $$

The ICC ranges from 0 to 1; if the unexplained measurement variability across the assessors for the same patient is small in comparison to the true variability across the patients, the ICC will be close to 1. We will also asses the agreement in area between the first and second assessor separately by depth grading where data allows using the outlined methods.

To enable the comparison of the three different necrosis outcome measures, we will categorise necrosis area measurements (agree/do not agree) for all patients if they are within ± 2.5% (total error margin of 5%) calculated agreement between assessors using Fleiss’ kappa statistic.

All this information will be assessed by the Trial Steering Committee to determine the most suitable and discriminating way in which to measure necrosis. To aid the decision process, a performance matrix [21] will be constructed (see Table 3). The performance of each measurement method against three key specified criterion will be completed.

**Table 3:** Performance matrix

Criteria	Outcome measure		
	Necrosis Y/N	Depth (SKIN)	Total necrosis area
Subjectivity of measurement (κ for the first and second clinical assessment)*			
Sample size required to demonstrate statistically significant difference between treatment and control arm (based on observed data)^†^			
Proportion of patients with observed response**			

*For total necrosis area, we include κ where area is assumed to be 0 mm^2^ when no necrosis present is recorded. ^†^For necrosis Y/N sample size will be determined for a two sample proportions test. For total area, sample size will be computed using non-parametric methods for non-normally distributed continuous data [22]. For necrosis depth sample size calculation for ordered categorical data will be performed using the observed proportions in each category [23]. **For total necrosis area, we will present the proportion of patients with an observed area response where area is assumed to be 0 mm^2^ where no necrosis present is recorded

The performance of each method of measurement will then be compared pairwise against all the others for each criterion. For each pairwise comparison, the superior method will be assigned a score of 1 for the associated criteria. Superiority will be declared for a higher κ statistic, lower sample size and higher proportion of patients with observed response. For ties (equal performance), we will assign each measurement a score of ½. For each pairwise measurement comparison, a total superiority score will be computed for each measurement by summing the number of criteria the measurement is superior for. The total number of criteria each measurement is superior for, summed over comparisons with each other measurement methods, will be computed to establish an overall priority performance ranking. This ranking can be used by the TSC to facilitate the decision process; however, it is not obligatory or necessarily prescriptive. Additional qualitative feedback from clinicians and patients on acceptability of measurement use will also be important to consider.

#### Analysis of secondary outcomes

Table 4 summarises the proposed analyses for the secondary outcomes. Additional post-operative outcomes assessed within the 30 follow-up period listed in Table 2 will be summarised by treatment group using suitable measures of central tendencies and variability for continuous data and frequencies and proportions for categorical data. No significance testing will be undertaken.

**Table 4:** Analysis of secondary outcomes

Secondary outcome	Analysis
Recruitment rate per month	We will present recruitment numbers by month and compute the average monthly recruitment figure.
Proportion of patients followed-up at 30 days	We will present the overall proportion of patients followed-up at 30 days and the proportion of patients followed-up at 30 days by treatment arm.
Compliance and adherence with heating protocol	We will present the frequency and proportion of patients complying with the allocated intervention as not adhering, one session, two sessions or fully adhering. We will present reasons for non-adherence where available. For each of the three heating sessions, we will present measures of central tendencies (mean and median) and variability (SD and IQR) for the time of occurrence of the heating application, the temperature of the water and duration of the heat supplication.
Length of stay in hospital following SSM/NSM (days)	Kaplan–Meier curves will be plotted by treatment arm for length of stay in hospital, where length of stay is defined as the difference in days between the date the patient was discharged from hospital, and the date they were admitted. A Cox proportional hazards model will be fitted to estimate the intervention effect on length of stay, adjusting for the minimisation variables. Adjusted time to event curves will be plotted. If the proportional hazard assumption is not deemed a reasonable assumption then an alternative method for adjusting the curve will be sought either through use of a different time-to-event model or stratification of the Kaplan–Meier curves [24, 25].
Proportion of patients with necrosis requiring surgical intervention	The proportion of patients with necrosis requiring surgical intervention will be estimated by treatment arm with 95% CI. The surgical intervention required will be summarised using frequencies and proportions by treatment arm.

#### Analysis of harm outcomes

The frequency of adverse events and the number of patients experiencing events will be tabulated by seriousness and relation to heating protocol (i.e. as adverse reaction (AR), serious adverse event (SAE) or serious adverse reaction (SAR)) and by treatment arm. Non-serious adverse events unrelated to the heating protocol (AE) are recorded in the medical notes only. All ARs, SAEs and SARs will be listed individual by intensity and treatment arm.

## Discussion

There is evidence that heat preconditioning could help improve the provision of skin-sparing mastectomy at minimal cost by reducing the occurrence of skin necrosis. PREHEAT is a randomised controlled feasibility study for a trial that will evaluate the effectiveness of local heat preconditioning with respect to its effects on wound healing after reconstructive breast surgery in patients with breast cancer. The primary aim of PREHEAT is to determine the best way to measure a skin necrosis to inform the design of the definitive larger trial of effectiveness.

This article describes the PREHEAT trial protocol and detailed statistical analysis plan. The trial sponsor is GSTT. Recruitment started on 9 March 2015 and completed on 7 March 2017. Database lock and data extraction for analysis is scheduled for November 2017 when all randomised patients will have completed the surgical procedure and follow-up period.

The statistical analysis plan includes a comparative framework to facilitate decision-making for selecting the most suitable and discriminating necrosis measure for use in a definitive randomised controlled trial. This pre-specified statistical analysis plan will increase the transparency of the data. The reporting of results from the outlined statistical analysis will comply with the Consolidated Standards of Reporting Trials (CONSORT) Extension to Pilot and Feasibility Trials [15, 16].

## Additional file

Additional file 1: SPIRIT checklist. (DOCX 42 kb)

## Abbreviations

AR: Adverse reaction
AUC: Area under the curve
BRCA: BReast CAncer susceptibility gene
CI: Confidence interval
CONSORT: Consolidated Standards of Reporting Trials
DIEP: Deep Inferior Epigastric Perforator flap
GSTT: Guy’s and St Thomas’ NHS Foundation Trust
ICC: Intraclass Correlation Coefficient
IQR: Interquartile range
ITT: Intention-to-treat
KCTU: Kings Clinical Trials Unit
LOS: Length of stay
NHS: National Health Service
NICE: National Institute of Clinical Excellence
NSM: Nipple-Sparing Mastectomy
PREHEAT: A randomised controlled feasibility trial of heat preconditioning on wound healing after reconstructive breast surgery among breast cancer patients
RCT: Randomised controlled trial
SAE: Serious adverse event
SAR: Serious adverse reaction
SD: Standard deviation
SKIN: Skin Ischaemia Necrosis Score to assess severity of mastectomy skin flap necrosis
SPIRIT: Standard Protocol Items: Recommendations for Interventional Trials
SSM: Skin-Sparing Mastectomy
TSC: Trial Steering Committee
